# The Need for Communication Skills Training in Oncology

**DOI:** 10.6004/jadpro.2013.4.3.4

**Published:** 2013-05-01

**Authors:** Dany Michaella Hilaire

**Affiliations:** From University of Massachusetts Boston, College of Nursing and Health Sciences, Boston, Massachusetts

## Abstract

Communication between health-care professionals and their patients is an element that can make all the difference in how those patients navigate their cancer journey. In a 2011 issue of the *Annals of Oncology*, Barth and Lannen reported on their systematic review and meta-analysis of the efficacy of communication skills training courses in oncology. In this issue of *JADPRO*, two authors share their perspectives on the findings of Barth and Lannen and what advanced practitioners (APs) can learn from their research.

In 2010, the US Department of Health and Human Services (HHS) launched Healthy People 2020, an initiative that provides science-based national objectives for improving the health of all Americans. Healthy People 2020 is the third such 10-year agenda to be launched by the HHS. The goals and objectives of Healthy People 2020 take into consideration the importance of effective communication between health-care providers and patients in improving patient outcomes (HHS, 2013).

One goal of this health agenda is to increase the proportion of persons who report that their health-care providers have satisfactory communication skills, listen carefully to them, explain medical information in a way they (the patients) are able to understand, as well as show respect for what their patients have to say (HHS, 2013). Effective communication between health-care professionals and patients generates information that is beneficial to patient outcomes and supports patient-centered care. Furthermore, effective communication translates to increased patient knowledge and shared understanding, increased adherence to treatment recommendations, and the adoption of healthier habits and self-care strategies (Epstein & Street, 2007). Health-care providers continue to report difficulty communicating with patients, particularly when they are faced with addressing health-care challenges such as cancer.

Beginning at the time of diagnosis of cancer and continuing throughout treatment and survivorship, effective communication is vitally important to patient outcomes. It may determine how well a patient copes with the disease process and treatment (Maguire & Pitceathly, 2002). Unfortunately, ineffective communication may increase levels of anxiety in patients and create uncertainty (Lamont & Christakis, 2001).

Health-care providers address psychosocial concerns through effective communication strategies. These may include validating and exploring emotional concerns and providing health information to decrease unfavorable emotions such as anxiety (Iwamitsu, Shimoda, Abe, Okawa, & Buck, 2005). When a health-care provider demonstrates good communication skills, cancer patients are more apt to adhere to treatment recommendations and have improved psychosocial functioning (Arora, 2003). In addition, effective communication may help alleviate anxiety and other psychosocial concerns of oncology patients (Kennedy Sheldon, 2005).

The psychosocial concerns of a cancer diagnosis may overpower patient inquiries about the disease process, especially if the patient has fatalistic views about cancer and cancer treatment (McWilliam, Brown, & Stewart, 2000). These concerns may also affect a patient’s well-being and quality of life (Arora et al., 2001). Health-care providers play an essential role in controlling and alleviating these negative sequelae, as patients continually seek their reassurance and support (Rose, 1990).

Communication skills training (CST) programs have been developed to improve both health-care providers’ abilities and levels of confidence in effectively communicating with their patients (Kennedy Sheldon, 2005; Kiss & Sollner, 2006). In oncology settings, CST programs may help health-care professionals demonstrate feelings of empathy (Barth & Lannen, 2011). Furthermore, this training may help health-care professionals address stressful and difficult situations for patients and their families (Barth & Lannen, 2011). The literature has also linked CST to improved quality of medical care and improved patient satisfaction (Roter, 2006; Griffin et al., 2004).

In 2011, Barth and Lannen published a systematic review assessing the efficacy of CST for health-care professionals. They included studies with either a control group with less training compared to the intervention group or a control group with no intervention at all. They examined the health-care professional and patient outcomes of CST. The health-care professional outcomes were communication behaviors and attitudes after training. Patient outcomes were satisfaction with the communication skills of health-care professionals, as assessed by surveys. The purpose of this review is to emphasize the importance of CST, particularly in oncology patients, underlining the findings by Barth and Lannen (2011).

## Methods of Systematic Review of CST Programs

To perform the systematic review, Barth and Lannen (2011) used four different databases: Central, PsycINFO, MEDLINE, and Embase. Studies included were controlled studies on CST in oncology focusing on training sessions addressing issues such as breaking bad news, dealing with emotional concerns of patients, and transitioning patients to palliative care. Participants had to be health-care professionals, including physicians, nurses, and social workers interacting with oncology patients. Training courses had to include some sort of active practice and had a minimum time requirement of 6 hours. Studies excluded from this review were those that emphasized recruitment strategies and recruitment of patients into clinical trials, shared decision-making, and genetic counseling.

Outcomes included communication behavior and attitudes of health-care professionals, as well as satisfaction of patients, focusing on outcomes specific to patients. The desired results of the three outcomes were transformed to *between group* effect sizes (ESs) by calculating standardized mean differences. An ES greater than zero was the desired effect. An ES of 0.20 to 0.50 was indicative of a low effect, an ES of 0.50 to 0.80 was indicative of a moderate effect, and an ES of greater than 0.80 was indicative of a large effect (Barth & Lannen, 2011).

## Results of Systematic Review

The initial search yielded 1,194 studies, but only 13 met the inclusion criteria and were included in the review. The length of training programs ranged from less than 24 hours to more than 36 hours. Training focused on improving basic communication skills, employing effective communication in delivering bad news, improving communication with family members, teaching skills on how to assess psychosocial concerns, and dealing with response to emotions. All of the studies used active practice through role-play. Health-care professional–specific outcomes in the studies included were assessed through video or audio recordings. Patient-specific outcomes were assessed through surveys assessing satisfaction with health-care professionals’ communication. In the review, few of the included studies (n = 4) assessed patient outcomes. Those that assessed patient outcomes had patients complete surveys focusing on their satisfaction with their physician’s communication.

Barth and Lannen (2011) calculated an ES of 0.54 (confidence interval = 0.27–0.81) in studies where there was no intervention for the control groups to demonstrate that CST improved communication skills. However, the duration of training courses influenced training efficacy. Patient simulation and real patient scenarios were used to assess the communication skills of health-care professionals after training. There was no difference in communication skills posttraining between patient simulations and real patient scenarios. Both techniques were effective in assessing communication skills of health-care professionals, which supports the assertion that skills acquired during training can easily be applied in the clinical setting.

## Limitations

Some limitations to this review included the small number of articles as well as the fact that outcomes measured were broad and not patient specific and that effectiveness of the CST programs was measured in the short term. There were a small number of studies that met the inclusion criteria (n = 13) for the review. The studies presented assessed health-care professional outcomes and patient outcomes; therefore, it may have been challenging to compare the different outcomes. There were only three specific outcomes addressed: communication and behaviors of health-care professionals after the training and patient satisfaction. This may be due to the lack of studies assessing how communication skills affect patient outcomes.

Furthermore, communication skills in the presented studies were measured short term. One can argue that a more effective assessment should be long term to see if the new skills are maintained over time. The review required CST with a minimum of 6 hours, which may have decreased the effect sizes of the trainings, as most training sessions lasted 2 to 3 days (Barth & Lannen, 2011).

## Discussion

Barth and Lannen’s (2011) systematic review adds information to the effectiveness of CST, particularly in oncology settings. It is important to note that Barth and Lannen (2011) took a multidisciplinary approach to communicating with oncology patients. They examined studies assessing the effectiveness of CST in all health-care professionals, not just doctors and nurses. Although the different studies included in this review focused on specific professionals, it is important to note that there are multiple disciplines involved in the care of oncology patients, and effective communication skills are essential for all health-care professionals.

The timing of CST programs for practicing clinicians is an important consideration. Ideally, CST should begin at the new graduate level and should continue throughout clinical practice in health care, as communication skills are oftentimes learned through experience. Combining the CST with clinical experiences allows health-care professionals to apply the skills acquired through training and may yield more effective, durable outcomes. Therefore, clearer goals need to be made about the long-term impact of CST. Despite the assessments conducted posttraining in these studies, Barth and Lannen (2011) supported the application of the skills acquired through training in the clinical setting to improve patient outcomes (Zachariae et al., 2003). Longitudinal assessments of communication skills may help researchers further understand areas where more training is needed and may influence the timing of delivery of specific CST training modules.

Communication skills training is needed for health-care professionals, especially for those dealing with vulnerable populations such as oncology patients and with issues regarding palliative care and end of life. The Oncotalk program emphasizes training on those tough topics that may be challenging for both the provider and the patient (Back et al., 2007). Back and colleagues reported positive results in using the Oncotalk program to train providers to develop effective communication skills when addressing difficult issues with patients (Back et al., 2007). Communication skills training may need to be reinforced through clinical practice, didactic workshops, and/or basic training. Barth and Lannen (2011) state that continuous training throughout the course of a health-care professional’s career may improve the communication skills acquired during clinical practice. Refreshing the communication skills of health-care professionals may yield more favorable patient outcomes.

As recommended by Healthy People 2020, there is a national goal of more effective communication between health-care professionals and patients. To assess more evidence of the effectiveness of CST, studies need to be designed to measure patient outcomes before and after CST interventions. In addition, further research needs to assess both patient outcomes and health-care provider outcomes. For example, researchers should not only assess health-care professionals’ perceptions of the effectiveness of their communication skills, but they should also assess patients’ perceptions of providers’ communication skills.

## Conclusion

Effective communication is nearly as important as medical intervention in promoting healthy outcomes for oncology patients. Further research assessing the effectiveness of CST in improving the communication of health-care professionals should focus on patient outcomes rather than health-care professionals’ rating of their perceived abilities. Longitudinal studies that follow health-care professionals over time may be more beneficial in assessing the effectiveness and durability of CST and also may help meet the Healthy People 2020 goal.
